# Indoor incense burning impacts cognitive functions and brain functional connectivity in community older adults

**DOI:** 10.1038/s41598-020-63568-6

**Published:** 2020-04-27

**Authors:** Adrian Wong, Wutao Lou, Kin-fai Ho, Brian Ka-fung Yiu, Shi Lin, Winnie Chiu-wing Chu, Jill Abrigo, Dustin Lee, Bonnie Yin-ka Lam, Lisa Wing-chi Au, Yannie Oi-yan Soo, Alexander Yuk-lun Lau, Timothy Chi-yui Kwok, Thomas Wai-hong Leung, Linda Chui-wa Lam, Ko Ho, Vincent Chung-tong Mok

**Affiliations:** 1 Department of Medicine and Therapeutics, The Chinese University of Hong Kong, Hong Kong SAR, China; 2 Therese Pei Fong Chow Research Centre for Prevention of Dementia, The Chinese University of Hong Kong, Hong Kong SAR, China; 3 Gerald Choa Neuroscience Centre, Lui Che Woo Institute of Innovative Medicine, The Chinese University of Hong Kong, Hong Kong SAR, China; 4 Department of Imaging and Interventional Radiology, The Chinese University of Hong Kong, Hong Kong SAR, China; 5 The Jockey Club School of Public Health and Primary Care, The Chinese University of Hong Kong, Hong Kong SAR, China; 6 Department of Psychiatry, Faculty of Medicine, The Chinese University of Hong Kong, Hong Kong SAR, China; 7 Stony Brook University School of Medicine, Stony Brook, New York, United States

**Keywords:** Cerebrovascular disorders, Neurodegenerative diseases, White matter disease, Risk factors, Diseases of the nervous system

## Abstract

To investigate (1) the effects of indoor incense burning upon cognition over 3 years; (2) the associations between indoor incense burning with the brain’s structure and functional connectivity of the default mode network (DMN); and (3) the interactions between indoor incense burning and vascular disease markers upon cognitive functions. Community older adults without stroke or dementia were recruited (n = 515). Indoor incense use was self-reported as having burnt incense at home ≥ weekly basis over the past 5 years. Detailed neuropsychological battery was administered at baseline (n = 227) and the Montreal Cognitive Assessment at baseline and year 3 (n = 515). MRI structural measures and functional connectivity of the DMN were recorded at baseline. Demographic and vascular risk factors and levels of outdoor pollutants were treated as covariates. Indoor incense burning was associated with reduced performance across multiple cognitive domains at baseline and year 3 as well as decreased connectivity in the DMN. It interacted with diabetes mellitus, hyperlipidemia and white matter hyperintensities to predict poorer cognitive performance. Indoor incense burning is (1) associated with poorer cognitive performance over 3 years; (2) related to decreased brain connectivity; and (3) it interacts with vascular disease to predispose poor cognitive performance.

## Introduction

**Figure 1:**
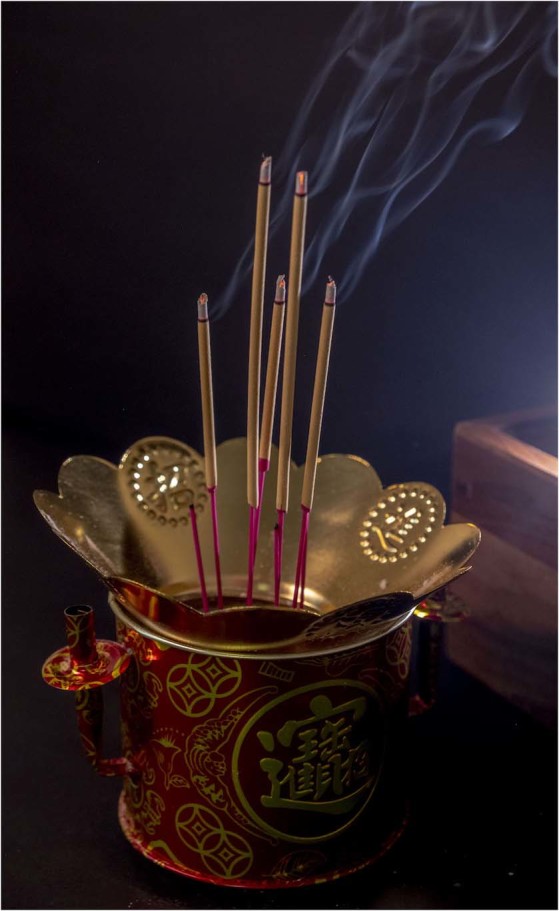
‘Joss sticks’ commonly burned in homes for ancestor worshipping.

Incense burning is a religious ritual commonly practised in many cultures and is popular among older adults. Incense comes in many forms, with ‘joss sticks’ being the commonest choice for home use (Fig. 1). Incense is made up of a mixture of fragrance materials and herbal, wood and adhesive powder^1^. When incense is burnt, pollutants including particulate matter (PM), carbon monoxide (CO), carbon dioxide (CO_2_), sulfur dioxide (SO_2_), nitrogen dioxide (NO_2_), volatile organic compounds, aldehydes and polycyclic aromatic hydrocarbons (PAHs) are released into the air^1,2^. Incense burning is considered a major source of indoor air pollution; the amount of PM generated by incense can be up to 4.5 times of that by cigarettes^2^. Incense smoke is associated with carcinogenicity, increased cardiovascular mortality and respiratory conditions^1,3–5^. Although there is currently a lack of published data showing a direct link between incense burning with cognitive and brain health, air pollution research suggests that pollutants emitted from incense smoke are associated with accelerated cognitive aging, intellectual decline and an increased risk for Alzheimer’s Disease (AD) and vascular dementia^6–11^. Moreover, long term exposure to air pollution is associated with smaller total brain volume and volume in prefrontal cortex, white matter and associations areas in frontal, temporal regions and corpus callosum. It is also related to the development of vascular pathology including covert brain infarcts, white matter hyperintensities (WMH, a marker of cerebral small vessel disease [SVD]), enlarged Virchow-Robin spaces, gliosis, atherosclerosis and a faster progression of carotid intima-medial thickness^9,10,12–14^ and increases risks of strokes and vascular cognitive impairment (VCI)^15^.

The objectives of this study are to investigate, in older adults free of stroke and dementia,

1) the effects of household indoor incense burning upon cognition over 3 years;

2) the effects of indoor incense burning upon structural MRI markers of SVD (WMH and lacune) and medial temporal lobe atrophy (MTLA), a proxy of AD, as well as functional connectivity of the default mode network (DMN); and

3) whether indoor incense burning moderates the relationships between vascular risk factors and structural imaging markers with cognitive functions.

## Methods

### Study design

Prospective, 3-year longitudinal case-controlled study.

### Participants

**Figure 2:**
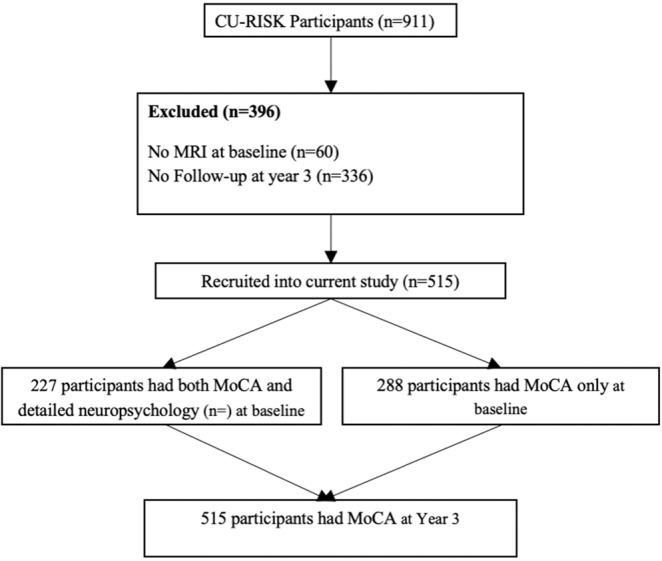
Flow diagram describing the sample recruitment and cognitive data collection.

Participants are stroke- and dementia-free community older adults recruited in the *Chinese University of Hong Kong RISK Index for Screening Subclinical Brain Lesions in Community-dwelling in Hong Kong* (CU-RISK Study). Inclusion criteria were (1) age ≥ 65 years; (2) community-dwelling and (3) written informed consent given. Exclusion criteria were (1) dementia at baseline, as defined by the locally validated education-adjusted cut-off scores on the Mini-mental State Examination^16^ or a clinical diagnosis of dementia; (2) history of stroke; (3) inadequately controlled psychiatric disorders; and (4) physical or sensory impediments hindering participation in cognitive testing. Baseline data collection took place between November 2011 and March 2016. A subset of 515 randomly selected participants received follow-up between October 2014 and December 2017. Flow diagram describing the sample recruitment and cognitive data collection is presented as Fig. 2. The CU-RISK study complies with the relevant guidelines in the Declaration of Helsinki and approval was granted from the Chinese University of Hong Kong – New Territories East Cluster Clinical Research Ethics Committee. This study is part of the Chinese University of Hong Kong “*Brain Health Brings Health*” programme.

### Recording of incense use habits and potential confounders

Incense use habit was recorded based on self-report as current regular practise of incense burning at home on a weekly or more frequent basis over the past 5 years. An apartment of total area between 200 to 800 square feet consisting of a living room and 1–3 bedrooms represents a typical indoor household environment in Hong Kong.

### Outdoor air pollutant levels

The general regional air quality data (AQD) published by Department of Environment Protection of the Government of Hong Kong Special Administrative Region was collected from a publicly accessible website (http://epic.epd.gov.hk/EPICDI/air/station/?lang=en) to measure the levels of 6 outdoor air pollutants: fine suspended particulates, NO_2_, O_3_, SO_2_, nitrogen oxides (NO) and respirable suspended particulates measured hourly over 13 districts of Hong Kong. The yearly averaged readings of the different pollutants measured in the station matched to the residential address of the individual participants were recorded.

### Cognitive assessment

Cognitive assessment was conducted by trained research assistants. The Montreal Cognitive Assessment (Hong Kong version; MoCA)^17^ covers cognitive domains including learning and memory, executive and visuospatial functions, language, attention, working memory, abstraction and orientation and is shown to be sensitive to mild cognitive dysfunction and cognitive change over time^18,19^. The total score is used as an index of global cognitive functions. In addition to the total score, domain scores for memory, executive functions, visuospatial functions, language and attention were calculated from a previously published method^20^. A subset of 274 participants also underwent detailed neuropsychological testing covering memory, executive functions/activation, language and visuospatial functions using the National Institute of Neurological Disorders and Stroke and Canadian Stroke Network VCI Harmonization 60-minute neuropsychology protocol at baseline^21,22^. Only the MoCA was administered at year 3 (Fig. 2).

### Neuroimaging

#### Image acquisition

Magnetic Resonance Imaging (MRI) of the brain was performed at baseline using a 3 Tesla Philips MRI scanner with an 8-channel head coil (Achieva TX, Philips Medical System, Best, The Netherlands). Structural MRI was obtained with T1W sequence (TR = 7.5 ms; TE = 3.5 ms; matrix = 240 × 240 × 285; voxel=1.04 mm × 1.04 mm × 0.6 mm^3^). Fluid-attenuated inversion recovery (FLAIR) images were also acquired for each participant with the following parameters: TR = 8000 ms, TE = 331 ms, inversion time=2400 ms, voxel size = 0.44 × 0.44 × 0.56 mm^3^. Functional images were available for 104 incense users and 96 age and education matched non-users. The resting-state functional MRI (fMRI) images were acquired with a T2-weighted gradient echo-planar imaging sequence: TR = 2050 ms, TE = 25 ms, flip angle = 90°, FOV = 205 × 205 mm^2^, slices = 47, voxel size=3.2 × 3.2 × 3.2 mm^3^. Participants were instructed to keep their eyes open and focus on a cross in the screen.

#### Data Pre-processing

MRI markers including WMH volume, lacune and MTLA were measured. The WMH was defined as hyperintensity as reflected on FLAIR image, according to the STRIVE standard^23^ and automatically segmented with manual correction where appropriate. WMH segmentation was performed based on a validated pipeline - coarse-to-fine detection of WMH using co-registered T1W and FLAIR^24^. Lacunae were defined according to the STRIVE standard and segmented in T1W images^23^. The number was counted as isolated regions automatically. MTLA was visually rated on T1W images using the Schelten’s scale^25^. fMRI imaging data were pre-processed using Statistical Parametric Mapping software version 12 (http://www.fil.ion.ucl.ac.uk/spm/). The first 10 volumes of each participant were discarded to allow for T1W equilibration effects. Then the functional images were slice-time corrected for timing offsets between different slices and realigned to the first image to correct for head motion between scans. The high-resolution T1W image was then co-registered to the mean of the corrected functional images. In order to improve the brain tissues segmentation in aging, a multi-channel segmentation approach in SPM 12 was used. The FLAIR image was coregistered to the T1W image, and the coregistered T1W and FLAIR images were combined underwent multi-channel segmentation to extract different tissues including gray matter, white matter and cerebrospinal fluid. A study-specific template was created using the DARTEL toolbox^26^. The functional images were then spatially normalized to the standard MNI space by using the nonlinear normalization parameters estimated by the DARTEL toolbox, resampled to 3 × 3 × 3 mm^3^ and spatially smoothed with a 6 mm full-width half-maximum Gaussian kernel. Finally, additional preprocessing steps were implemented to the normalized function images to eliminate the effect of low-frequency drifts and physiological noise, which included removing linear trends, temporally band-pass filtering (0.01–0.1 Hz) and regressing out several nuisance signals (six head-motion profiles, the averaged signals from white matter, cerebrospinal fluid, and the whole brain and the first derivatives thereof)^27^.

#### Functional connectivity analysis in the DMN

The DMN was identified using a seed-based correlation approach. First, a 5-mm-radius sphere seed centred on the posterior cingulate cortex (MNI coordinates: -7, -43, 33) was selected^28^. The mean BOLD time series of the seed was then extracted and correlated with every voxel of the whole brain using Pearson correlation. The correlation coefficients were then converted to z-scores using Fisher’s r-to-z transformation. A one-sample t-test was used to identify the DMN for each group, the p-value threshold was set to *p* < 0.05 (false discovery rate correction (FDR) for multiple comparisons, voxel size > 30). Then, analysis of covariance (ANCOVA) was used to evaluate the group differences between the incense users and non-users in the DMN with age, sex and years of education as covariates. The significance threshold was set to *p* < 0.05 (FDR corrected, voxel size > 30).

### Statistical analysis

Statistical analysis was performed using IBM SPSS version 21.0. Statistical significance was determined at *p* < 0.05 for all analyses. Participants were categorized into those who reported having regularly burned incense (users) and who did not (non-users). Group comparisons on demographic and clinical data were conducted using independent sample t tests or Chi-squared tests as appropriate. ANCOVA was used to compare the cognitive performance between the groups at baseline and year 3. The group effect size on cognitive outcomes was measured by the Cohen’s *d* statistic, with 0.2, 0.5, and ≥0.8 indicating small, medium and large effects, respectively^29^. Furthermore, a repeated measure ANCOVA was used and a group x time interaction term was calculated to test whether incense users and non-users experienced a different cognitive trajectory over 3 years as measured by the MoCA Total score. Linear regression models were constructed to examine the associations between incense burning and cognitive functions at baseline and year 3 and the change in MoCA Total score over 3 years. Variables selected as covariates were demographic factors (age and years of education), vascular risk factors (hypertension, diabetes mellitus [DM] and hyperlipidemia, current smoking, current alcohol drinking) and 6 types of outdoor air pollutants levels. Table 1 enlists the cognitive and imaging markers used as outcomes in this study.

**Table 1:** List of cognitive and imaging markers used as outcomes of the study.

Variable	Outcome
**Cognitive health markers**	
*Cognitive functions at baseline*	
MoCA Total	Global Cognition
MoCA Executive Domain	Executive Functions
CTT1	
CTT2	
SDMT	
Verbal Fluency	
MoCA Language Domain	Language
mBNT	
MoCA Visuospatial Domain	Visuospatial functions
RCFT Copy	
MoCA Attention Domain	Attention
MoCA Memory Domain	Memory
HKLLT Learning	
HKLLT 30-min delayed recall	
HKLLT 30-min delayed recognition	
RCFT 30-min delayed recall^*^	
*Cognitive Performance at year 3*	
MoCA Total score	Global Cognition
MoCA Executive Domain	Executive Functions
MoCA Language Domain	Language
MoCA Visuospatial Domain	Visuospatial functions
MoCA Attention Domain	Attention
MoCA Memory Domain	Memory
**Brain health markers**	
*Structural imaging measures*	
MTLA rating	Alzheimer’s Disease marker
Lacunar infarcts number	Cerebral small vessel disease markers
WMH volume (mm^3^)	
*Functional imaging measures*	
Middle Temporal Gyrus (R)	Functional disconnection of the default mode network
Medial Frontal Gyrus	
Precuneus	
Angular (L)	

Abbreviations: mBNT = modified Boston Naming Test; CTT = Colour Trails Test; HKLLT = Hong Kong List Learning Test; MoCA = Montreal Cognitive Assessment; MTLA = Medial Temporal Lobe Atrophy Rating (Schelten’s Scale); RCFT = Rey Complex Figure Test; SDMT = Symbol Digit Modalities Test; WMH = White Matter Hyperintensities.

Continuous variables were examined first for collinearity. As significant collinearity was found between fine suspended particulates and respirable suspended particulates and between NO_2_ and nitrogen oxides, only the results based on fine suspended particulates and NO_2_ are presented. Two-way ANCOVA was conducted to examine the interactions between incense burning with vascular risk factors or structural imaging markers upon MoCA Total score while controlling for demographic and other vascular risk factors. For imaging markers, participants were classified into low and high WMH levels by median split at 3.74mm^3^ based on data of the whole sample. MTLA was determined as ratings ≥2 on the Schelten’s scale^25^.

In view of the group difference in age and year of education in the whole sample, propensity score was used to match incense users and non-users with age and years of education as matching covariates for functional imaging data analysis and as sensitivity analysis for structural imaging data. Each incense user was matched to one non-user without replacement. The distributions of matching covariates between groups were compared with the matched sample to ensure a balanced matching. Inverse probability of treatment weighting (IPTW) using the propensity score was used to create a synthetic sample such that the baseline covariates were independent of grouping.

### Ethics approval and consent to participate

The Chinese University of Hong Kong – New Territories East Cluster Clinical Research. Ethics Committee approved the CU-RISK study. Informed consent was obtained from the study participants.

## Results

Five hundred and fifteen participants were recruited. Compared to non-users, users were 1.1 years older, had 2.7 years less education and were less likely to have hypertension. (Table 2). Exposure to outdoor pollutants was similar between groups. The 227 participants with detailed neuropsychological testing were similar in age and sex compared to those without. Participants with detailed testing had slightly lower MoCA Total score (mean difference −1.42 [3.81]) and less education (mean difference in years −1.84 [4.81]) compared to those without detailed testing. The interval between baseline and follow-up was 38.3 (4.1) months. Descriptive statistics and effect size estimates (where applicable) are presented in Table 2.

**Table 2:** Comparisons between incense users and non-users.

	Non-users	Users	*p*		Missing data (%)
N	359	156			
Demographic characteristics					
Age in years	70.7 ± 4.4	71.8 ± 4.8	0.009		0
Female, n (%)	63.5	66	0.584		0
Education in years	8.9 ± 4.8	6.2 ± 4.4	<0.001		0
Vascular risk factors					
Hypertension, n (%)	151 (42.1)	49 (31.4)	0.023		0
Hyperlipidemia, n (%)	243 (67.7)	101 (64.7)	0.514		0
Diabetes mellitus, n (%)	282 (78.6)	115 (73.7)	0.230		0
Current Smoking, n (%)	9 (2.5)	7 (4.5)	0.234		0
Current Alcohol drinking n (%)	57 (15.9)	22 (14.1)	0.608		0
Cognitive Performance at baseline					
*Global Cognition*			Cohen’s *d*		
MoCA Total	23.6 ± 3.4	21.2 ± 4.2	<0.001	0.63	0
*Executive Functions*					
MoCA Executive Domain	3.0 ± 1.2	2.4 ± 1.2	0.013	0.50	0
CTT1^*^	65.2 ± 25.3	80.4 ± 38.1	0.158	0.47	1
CTT2^*^	127.6 ± 41.1	151.6 ± 65.3	0.102	0.45	4
SDMT	32.3 ± 10.5	25.9 ± 11.5	0.025	0.58	0
Verbal Fluency	16.3 ± 4.3	14.5 ± 4.1	0.024	0.43	0
*Language*					
MoCA Language Domain	4.6 ± 0.7	4.3 ± 0.9	0.073	0.38	0
mBNT^*^	14.1 ± 1.3	13.8 ± 1.5	0.879	0.21	0
*Visuospatial functions*					
MoCA Visuospatial Domain	3.2 ± 0.9	2.7 ± 1.1	0.012	0.50	0
RCFT Copy^*^	26.1 ± 7.4	23.0 ± 10.2	0.230	0.35	38
*Attention*					
MoCA Attention Domain	3.6 ± 0.7	3.5 ± 0.8	0.375	0.13	0
*Memory*					
MoCA Memory Domain	9.2 ± 1.8	8.4 ± 1.7	0.009	0.64	0
HKLLT Learning^*^	21.8 ± 6.6	19.5 ± 6.3	0.557	0.36	0
HKLLT 30-min delayed recall^*^	6.7 ± 3.3	5.6 ± 3.2	0.272	0.36	0
HKLLT 30-min delayed recognition^*^	13.4 ± 2.1	12.8 ± 2.8	0.955	0.25	0
RCFT 30-min delayed recall^*^	11.3 ± 6.3	7.2 ± 6.1	0.003	0.66	1
Cognitive Performance at year 3					
MoCA Total score	23.2 ± 4.0	20.8 ± 4.5	0.006	0.57	0
MoCA Executive Domain	3.1 ± 1.2	2.8 ± 1.2	0.510	0.29	0
MoCA Language Domain	4.5 ± 0.7	4.4 ± 0.9	0.672	0.11	0
MoCA Visuospatial Domain	3.1 ± 0.9	2.6 ± 1.1	0.013	0.50	0
MoCA Attention Domain	3.5 ± 0.7	3.4 ± 0.9	0.954	0.13	0
MoCA Memory Domain	9.0 ± 2.1	7.7 ± 2.0	<0.001	0.63	0
Structural imaging measures					
*All participants*					
MTLA rating	0.9 ± 0.8	1.0 ± 0.9	0.828		0
Lacunar infarcts number	0.8 ± 1.7	1.1 ± 2.6	0.999		0
WMH volume (mm^3^)	5.6 ± 6.4	6.2 ± 7.2	0.952		2
*Age and education matched groups; n* = 200					
MTLA rating	0.9 ± 0.7	1.0 ± 1.0	0.428		0
Lacunar infarcts number	0.8 ± 1.8	0.9 ± 1.6	0.893		0
WMH volume (mm^3^)	6.0 ± 6.0	6.1 ± 6.8	0.889		1
Functional imaging measures					
Functional Connectivity of Default Mode Network (seeds −7,−43,33); values in brain regions with significant difference (Fisher’s r-to-z transformed value) shown					
Middle Temporal Gyrus (R)	0.2 ± 0.2	0.1 ± 0.2	<0.001		0
Medial Frontal Gyrus	0.3 ± 0.2	0.2 ± 0.2	<0.001		0
Precuneus	0.7 ± 0.2	0.5 ± 0.2	<0.001		0
Angular (L)	0.6 ± 0.2	0.4 ± 0.2	<0.001		0
Yearly averaged level (in µg/m^3^) of outdoor air pollutants at reported residential address in health record					
Fine Suspended Particulates	30.2 ± 3.6	30.0 ± 2.7	0.571		0
Nitrogen Dioxide (NO_2_)	58.9 ± 9.6	60.1 ± 9.8	0.223		0
Ozone (O_3_)	36.6 ± 7.5	35.8 ± 7.5	0.271		0
Sulphur Dioxide (SO_2_)	12.3 ± 3.2	13.1 ± 3.2	0.012		0
Nitrogen Oxides (NO)	107.2 ± 23.5	111.1 ± 23.8	0.103		14
Respirable Suspended Particulates	44.3 ± 4.2	44.3 ± 3.1	0.978		0

Abbreviations: mBNT = modified Boston Naming Test; CTT = Colour Trails Test; HKLLT = Hong Kong List Learning Test; MoCA = Montreal Cognitive Assessment; MTLA = Medial Temporal Lobe Atrophy Rating (Schelten’s Scale); NO = Nitrogen Oxides; NO_2_ = Nitrogen Dioxide; O_3_ = Ozone; RCFT = Rey Complex Figure Test; SDMT = Symbol Digit Modalities Test; SO_2_ = Sulphur Dioxide; WMH = White Matter Hyperintensities.

Notes: Values are presented as mean ± standard deviation or % as appropriate.

Cognitive scores are compared using analysis of covariance adjusted for age, education year, hypertension, diabetes mellitus, hyperlipidemia, current smoking, current alcohol drinking and yearly average levels of fine suspended particulates, nitrogen dioxide, ozone and sulphur dioxide collected from the general regional air quality data (AQD) published by Department of Environment Protection of the Government of Hong Kong Special Administrative Region.

^*^Administered as part of a detailed neuropsychology assessment in a subset of participants (n = 227).

### Cognitive outcomes

#### Baseline

Compared to non-users, incense users had significantly poorer performance in measures of global cognition (MoCA Total score), executive functions (Symbol Digit Modalities Test [SDMT] and Verbal Fluency), visuospatial functions (MoCA Visuospatial Domain score) and memory (MoCA Memory Domain Score and Rey Complex Figure Test [RCFT] 30-minute delayed recall) after adjustment for age, education year, vascular risk factors and levels of outdoor pollutants. Among the measures with significant group difference, effect size (Cohen’ *d*) ranged between 0.43 for Verbal Fluency and 0.66 for RCFT 30-minute delayed recall. The groups did not differ in language and attention measures. (Table 2).

#### Year 3

The MoCA was administered at year 3, significant group difference persisted for MoCA Total score, MoCA Visuospatial Domain score and MoCA Memory Domain score (Table 2). The group x time interaction was not statistically significant (*p* = 0.732), indicating that the trajectory of change in MoCA Total score between baseline and year 3 did not differ between incense users and non-users.

### Associations between incense burning and cognitive outcomes at baseline and year 3

Linear regression showed that incense burning negatively contributed to performance on global cognition (MoCA Total score), executive functions (SDMT and Verbal Fluency), visuospatial functions (MoCA Visuospatial Domain score and RCFT copy) and memory (MoCA Memory Domain Score and RCFT 30-minute Delayed Recall) at baseline. At year 3, incense burning was negatively associated with performance on global cognition (MoCA Total score), executive functions (Verbal Fluency), visuospatial functions (MoCA Visuospatial Domain score) and memory (MoCA Memory Domain score). Table 3 shows the results of the linear regression models. Incense burning was not associated with change in MoCA Total score over 3 years (β = 0.021, *p* = 0.651).

**Table 3:** Linear regression models for associations between indoor incense burning and cognitive functions.

Outcome	Baseline			Year 3		
	β	*p*	Adjusted R^2^	β	*p*	Adjusted R^2^
*Global Cognition*						
MoCA Total	−0.13	<0.001	0.396	−0.11	0.004	0.333
*Executive Functions*						
MoCA Executive Domain	−0.10	0.009	0.293	0.03	0.510	0.293
CTT1	0.08	0.178	0.256	0.01	0.937	0.243
CTT2	0.10	0.110	0.205	0.02	0.752	0.186
SDMT	−0.07	0.023	0.506	−0.05	0.108	0.472
Verbal Fluency Test	−0.10	0.016	0.129	−0.10	0.017	0.143
*Language Functions*						
MoCA Language Domain	−0.08	0.074	0.115	0.02	0.672	0.102
mBNT	−0.01	0.916	0.088	0.05	0.537	0.110
*Visuospatial Functions*						
MoCA Visuospatial Domain	−0.10	0.011	0.296	−0.10	0.014	0.281
RCFT Copy	−0.17	0.042	0.022	0.07	0.334	0.239
*Attention Functions*						
MoCA Attention Domain	−0.04	0.376	0.080	0.00	0.954	0.139
*Memory Functions*						
MoCA Memory Domain	−0.11	0.006	0.182	−0.19	<0.001	0.154
HKLLT learning score	−0.04	0.524	0.268	0.11	0.126	0.219
HKLLT 30-min delayed recall	−0.08	0.226	0.177	0.05	0.484	0.102
HKLLT 30-min delayed recognition	−0.01	0.838	0.130	0.01	0.848	0.160
RCFT 30-min delayed recall	−0.16	0.007	0.288	−0.05	0.464	0.238

Abbreviations: mBNT = modified Boston Naming Test; CTT = Colour Trails Test; HKLLT = Hong Kong List Learning Test; MoCA = Montreal Cognitive Assessment; RCFT = Rey Complex Figure Test; SDMT = Symbol Digit Modalities Test. Models adjusted for age, education year, hypertension, diabetes mellitus, hyperlipidemia, current smoking, current alcohol drinking and yearly average levels of outdoor air pollutants

### Neuroimaging outcomes

#### Structural MRI

No group difference was found in WMH volume, lacune count and MTLA. Sensitivity analysis conducted in the propensity matched age- and education-matched sample (n = 200, incense users n = 104, non-users n = 96) yielded essentially the same results (Table 2).

#### Resting-state fMRI

**Figure 3:**
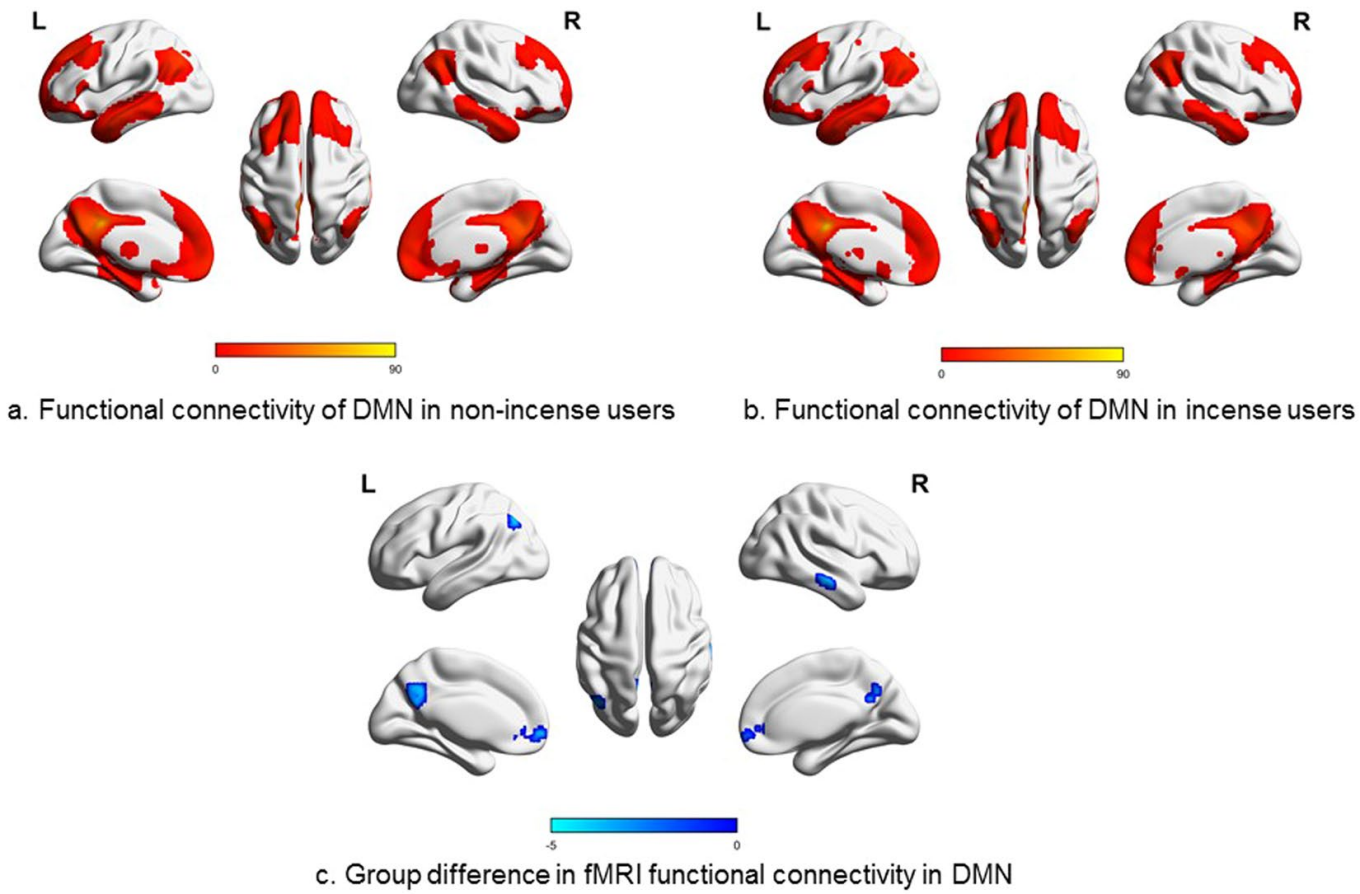
Results of fMRI seed-based functional connectivity in the DMN. One-sample t-test was used to identify the DMN with p-value threshold set to *p* < 0.05 with FDR correction (voxel size >30) in non-incense users (**a**) and incense users (**b**). Two-sample t-test was used to evaluate the group differences. Compared to non-users, self-report incense users showed significantly decreased connectivity in the Precuneus, Medial Frontal Gyrus, left Angular and right Middle Temporal Gyrus. The blue indicates decreased connectivity (*p* < 0.05) in the incense group (**c**).

Functional connectivity of the DMN of the non-users and users are shown in Fig. 3a.b, respectively. Significant DMN clusters of both groups were mainly identified in the superior/medial frontal gyrus, precuneus, and middle temporal gyrus. Incense users had significantly decreased connectivity in the precuneus, medial frontal gyrus, left angular and right middle temporal gyrus (Fig. 3c). Table 2 shows the numerical group comparison of the averaged functional connectivity values in the brain regions with significant group differences.

#### Interactions between incense burning with vascular risk factors and structural imaging markers upon cognitive performance

**Figure 4:**
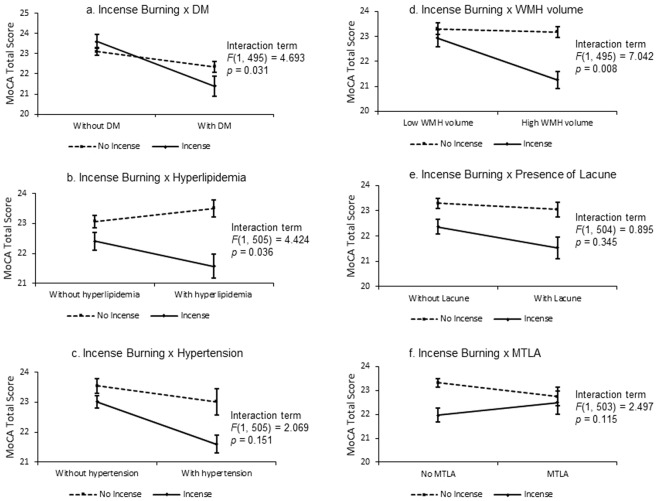
Interaction between incense burning with vascular risk factors and structural neuroimaging measures on MoCA Total score at baseline. Significant interactions were found between incense burning with DM (**a**), hyperlipidemia **(b**) and WMH (**d**) volume upon the MoCA Total score.

Significant interactions were observed between incense burning with DM (*p* = 0.031), hyperlipidemia (*p* = 0.036) and WMH volume (*p* = 0.008) on baseline MoCA Total score, indicating that incense burning had significantly more negative impact upon global cognition in the presence of DM, hyperlipidemia and increasing WMH volume. No interaction was found between incense burning with hypertension, the presence of lacune or significant MTLA on the MoCA Total score (Fig. 4).

## Discussion

This study shows that regular indoor incense burning is associated with poorer performance across multiple cognitive domains over 3 years. However, indoor incense burning was not associated with a more rapid decline in cognitive functions as measured by the MoCA Total score. Furthermore, although participants who practised indoor incense burning did not have more structural brain changes in terms of SVD lesions measured as WMH or lacune or MTLA, which is an imaging biomarker for AD, a subtle impact of incense burning upon the brain was evident on the level of functional connectivity of the DMN. The DMN is active at rest and during introspective, self-referential processing. The proper switching between the DMN and active brain networks is responsible for a variety of cognitive processes and its dysregulation is associated with a number of neurological and psychiatric conditions including attention deficit hyperactivity disorder^30^, autism spectrum disorder^31^, schizophrenia^32^, mild cognitive impairment^33^, AD as well as the progression of mild cognitive impairment into dementia^34^. This study suggests that indoor incense burning induces functional changes in the brain that may reduce cognitive resilience through functional connectivity alternation and thereby increases vulnerability for future cognitive decline, a hypothesis to be tested in future longitudinal studies. Moreover, although incense users did not have a higher frequency of DM, hyperlipidemia or WMH, incense burning appeared to interact with these vascular diseases to predispose poor cognitive functioning, highlighting the potential role of incense burning as a risk factor for VCI. Given the high prevalence of vascular burden among older adults, indoor incense burning should be practised with caution in this vulnerable population.

The precise mechanisms underlying the relationships between indoor incense burning with cognitive and brain changes need further investigations. Previous studies have reported that a wide range of air pollutants including ultrafine particles, PM, and volatile organic compounds produced from incense burning are detrimental to the vascular health^35^. Studies have demonstrated the capacity of incense smoke to induce oxidative stress and inflammation^36–39^, which may lead to significantly reduced vascular nitric oxide levels and increased levels of endothelin-1 and inflammatory mediators, such as granulocyte-macrophage-colony stimulating factor. The unregulated levels of these mediators would destruct the vascular vasodilation and constriction^40,41^, thereby promoting vascular dysfunction. It was suggested the water-soluble factions in particulate matter such as the transition metals with redox potential can also accelerate the process in membrane lipid peroxidation, which trigger endothelial cell mutations^42^. Moreover, the greater surface-to-mass ratio of ultrafine particles and PM can further increase the contacts with cells and enhance the damages, which may eventually lead to hemorrhagic events in blood vessels^43^.

To our knowledge, this is the first study to demonstrate the adverse effects of indoor household incense burning upon cognitive functions and brain health. The strengths of this study include a relatively large and well-defined community sample with a combination of cognitive, structural and functional imaging data and longitudinal follow-up. Also, the influence of regionally measured levels of a variety of outdoor air pollutants was taken into account as an important confounder. This study has a number of limitations. First, as incense use habit was evaluated from self-report, its accuracy might have been subjected to reporting bias. Second, although the common practise is to burn incense one to three times a day, each time using one to three incense sticks, details of incense use habits and home environment pertinent to the amount of exposure, such as the frequency of incense burning, the type and number of incense sticks used, indoor airflow variables, the specific location at home where the incense was burned and concomitant sources of indoor air pollutions, were not recorded. Third, past exposure to incense smoke was not assessed. However, it is safe to assume that these older participants have practised incense burning as religious rituals for many decades. Fourth, without real-time indoor air quality monitoring, the contributory roles of the various pollutants contained in incense smoke upon cognitive and neuroimaging outcomes could not be measured precisely. Last but not least, detailed neuropsychology and neuroimaging were not repeated at longitudinal follow up. Despite these limitations, this study has identified indoor incense burning as a novel and easily modifiable risk factor for adverse cognitive and brain health.

## Conclusions

Indoor incense burning may be detrimental to cognitive and brain health in community older adults. The present findings have potentially far-reaching public health implications for cultures with widespread home use of incense as worshipping rituals across cultures with a combined population size of 3.2 billion globally. Such implications are particularly relevant for older adults given the potential links between indoor incense burning and VCI. Results of this study call for safer practise of indoor incense burning, for example, by avoiding burning incense indoor or using safer incense alternatives.

## Data Availability

The dataset analysed during the current study is available from the corresponding author on reasonable request.
